# GDF15 modulates aging-associated adaptions in the mechanoresponse of periodontal ligament fibroblasts

**DOI:** 10.1007/s00056-025-00600-2

**Published:** 2025-07-15

**Authors:** Judit Symmank, Elena Rieger, Christoph-Ludwig Hennig, Annika Döding, Ulrike Schulze-Späte, Collin Jacobs

**Affiliations:** 1 Department of Orthodontics, Jena University Hospital, Jena, Germany; 2 Section of Geriodontics, Department of Conservative Dentistry and Periodontics, Jena University Hospital, Jena, Germany

**Keywords:** Orthodontic tooth movement, Aging model, Growth differentiation factor 15, Bone Remodeling, Hyperinflammation, Kieferorthopädische Zahnbewegung, Alterungsmodell, Wachstumsdifferenzierungsfaktor 15, Knochenremodeling, Hyperinflammation

## Abstract

**Purpose:**

This *in vitro* study aimed to examine aging-associated adaptations in the mechanoresponse of periodontal ligament fibroblasts (PdLFs) and explore a potential regulatory role of growth differentiation factor 15 (GDF15).

**Methods:**

Replicative senescence was induced in cultured human PdLFs (hPdLFs) by repeated enzymatic subcultivation as a valid *in vitro* aging model. The senescent phenotype was assessed by analyzing cellular morphology, proliferative capacity, and senescence marker expression. Furthermore, GDF15 levels were measured and subsequently modulated by small interfering RNA(siRNA)-mediated knockdown and by ponsegromab, a novel GDF15-neutralizing antibody. Tensile and compressive forces were applied using BioFlex® Culture Plates (FLEXCELL®, Dunn Labortechnik, Asbach, Germany) and sterile glass plates, respectively. The mechanoresponses of hPdLFs were characterized by analyzing pro-inflammatory cytokine levels and the activation of immune cells as well as of bone-remodeling osteoblasts and osteoclasts.

**Results:**

Aged hPdLFs exhibited decreased proliferation, increased β‑galactosidase activity, and morphological changes indicative of cellular senescence. Elevated baseline and force-induced intra- and extracellular GDF15 levels were observed in aged hPdLFs correlating with enhanced pro-inflammatory cytokine expression and immune cell activation. While aged hPdLFs showed reduced activation of osteoblasts in response to tensile forces, compressive forces led to increased osteoclast activation. Remarkably, modulating intra- and extracellular GDF15 levels partially restored the deregulated activation of bone-remodeling cells.

**Conclusion:**

Aging altered the mechanobiological response of PdL fibroblasts by promoting a hyperinflammatory microenvironment and shifting bone remodeling towards degenerative processes. Senescence-associated increases in GDF15 contributed to these changes by force-dependent intra- and extracellular signaling pathways. Targeting GDF15 could offer a therapeutic potential to optimize bone remodeling and improve orthodontic care for the elderly.

## Abstract (de)

**Zielsetzung:**

Ziel dieser *In-vitro-*Studie war es, altersassoziierte Anpassungen in der Mechanoreaktion von parodontalen Ligamentfibroblasten (PdLFs) zu untersuchen und eine mögliche regulatorische Rolle des „growth differentiation factor 15“ (GDF15) zu analysieren.

**Methoden:**

Replikative Seneszenz wurde in kultivierten menschlichen PdLFs (hPdLFs) durch wiederholte enzymatische Subkultivierung als valides *In-vitro*-Alterungsmodell induziert. Ihr seneszenter Phänotyp wurde durch Analyse der Zellmorphologie, der Proliferationsfähigkeit und der Expression von Seneszenzmarkern ermittelt. Darüber hinaus wurden die GDF15-Konzentrationen analysiert und anschließend durch „small interfering“ RNA (siRNA)-vermittelten Knockdown und Ponsegromab, einen innovativen GDF15-neutralisierenden Antikörper, moduliert. Zug- und Druckkräfte wurden mittels BioFlex® Kulturplatten (FLEXCELL®, Dunn Labortechnik, Asbach, Deutschland) bzw. durch sterile Glasplatten erzeugt. Die mechanischen Reaktionen der hPdLFs wurden durch die Analyse des Levels an pro-inflammatorischen Zytokinen und der Aktivierung von Immunzellen sowie von knochenumbauenden Osteoblasten und Osteoklasten charakterisiert.

**Ergebnisse:**

Gealterte PdLF zeigten eine verringerte Proliferation, erhöhte β‑Galaktosidase-Aktivität und morphologische Veränderungen, die auf zelluläre Seneszenz hinweisen. Erhöhte Ausgangswerte und kraftinduzierte intra- und extrazelluläre GDF15-Konzentrationen wurden in gealterten hPdLFs beobachtet, die mit einer verstärkten Expression pro-inflammatorischer Zytokine und der Aktivierung von Immunzellen korrelierten. Während die Aktivierung von Osteoblasten bei gealterten hPdLFs als Reaktion auf Zugkräfte reduziert war, führten Druckkräfte zu einer erhöhten Aktivierung von Osteoklasten. Interessanterweise kompensierte die Modulation der intra- und extrazellulären GDF15-Konzentration teilweise die deregulierte Aktivierung von Knochenumbauzellen.

**Schlussfolgerung:**

Alterungsprozesse veränderten die Mechanoreaktion von PdL-Fibroblasten, indem sie eine hyperinflammatorische Mikroumgebung förderten und den Knochenumbau in Richtung degenerativer Prozesse verschoben. Der seneszenzassoziierte Anstieg von GDF15 trug über kraftabhängige intra- und extrazelluläre Signalwege zu diesen Veränderungen bei. Eine zielgerichtete Modulation von GDF15 könnte ein therapeutisches Potenzial zur Optimierung des Knochenumbaus und zur Verbesserung der kieferorthopädischen Versorgung älterer Menschen bieten.

## Introduction

In correlation with increasing life expectancy and growing health consciousness specifically among the elderly, the number of older patients undergoing orthodontic treatment is steadily rising. This in turn requires adjustments of the orthodontic treatment strategy to the anatomical and tissue-specific changes that occur with aging, as these potentially extend beyond differences observed between adolescents and adults. Clinically, aging is associated with an increased risk of periodontal inflammation, root resorption, attachment loss and pain sensation [1]. Notably, aging specifically impacts the functionality of the periodontal ligament (PdL; [2]), which is critical for orthodontic tooth movement (OTM). PdL fibroblasts (PdLF), the predominant cell type within the ligament, reside between the tooth and the alveolar and modulate tissues and bone remodeling processes induced by orthodontic forces that enable tooth movement [3]. These mechanobiological processes include the initiation of force-induced inflammatory responses and the activation of bone remodeling cells, namely osteoblasts and osteoclasts, respectively [4–8].

Theoretically, two main profiles of PdL responses can be distinguished: one caused by tensile forces in areas where the PdL is stretched; the other by compressive forces in those areas where the PdL is compressed [9–11]. While tensile forces promote an anti-inflammatory PdL response and activation of bone-forming osteoblasts, compressive forces rather support a pro-inflammatory microenvironment and activation of bone-resorbing osteoclasts [8, 10]. This allows alveolar bone resorption and tooth movement within the direction of the applied force.

As a stress-responsive marker, growth differentiation factor 15 (GDF15) was recently reported as a pro-inflammatory modulator in the mechanoresponse of PdL cells [7, 12–16]. Up-regulated by both tensile and compressive forces, GDF15 appears to act via distinct intra- and extracellular signaling pathways mediating inflammatory responses and activation of bone remodeling cells. Elevated serum levels of GDF15 have been reported during aging [17], and prolonged exposure has been linked to an altered mechanoresponse of PdL fibroblasts [13]. However, the potential effects of the aging-associated GDF15 up-regulation on the response of PdL cells to mechanical stress are currently unknown.

In addition to its influence on GDF15, aging may also alter the inflammatory microenvironment in the mechanically stimulated PdL due to the accompanying senescence-associated secretory phenotype (SASP; [18]). Several cytokines including interleukins (IL), such as IL‑6 and IL-1β, are markedly elevated in aging, contributing to a chronic low-grade pro-inflammatory state [19]. Moreover, the proliferative and osteogenic differentiation capacity of PdL cells has been reported to decline with age [20]. Alveolar bone formation therefore appears to be compromised with aging. By contrast, increased osteoclast activity in alveolar bone areas adjacent to the PdL has been reported in studies on aged mice [21]. Thus, alveolar bone resorption may accelerate with increasing age.

Activation of immature osteoclast progenitors is triggered by the binding of the receptor activator of NF-κB ligand (RANKL) to its membrane receptor RANK [22]. However, RANKL is also bound by secreted osteoprotegerin (OPG), which thus exerts an inhibitory effect on osteoclastogenesis by acting as a decoy receptor. Both RANKL and OPG are expressed by PdLFs and secreted in a stress-dependent manner [23]. Therefore, compressive forces promote the secretion of RANKL and attenuate that of OPG, resulting in an increased RANKL/OPG ratio and the activation of osteoclast differentiation and alveolar bone degradation. In addition, RANKL also activates odontoclasts, which promote the degradation of dentin, cementum, and enamel [24]. Hyperactivity of osteoclasts and odontoclasts can contribute to the risks associated with orthodontic treatments, including tooth root resorption and alveolar bone loss [24].

In general, an aging-associated increase in the RANKL/OPG ratio has been observed, which appears to correlate with aging-associated bone loss [25, 26]. Regarding age-related changes during orthodontic treatments, a comparison between adolescent and adult patients interestingly revealed a lower RANKL/OPG ratio and attenuated tooth movement at early treatment stages in adults [27]. This was also shown by studies in rats, which reported more dynamic bone resorption activity in juveniles compared with adults [28, 29]. However, none of these studies examined aged individuals, which were classified according to general classification criteria starting at an age of about 60 years in humans or 18 months in mice or rats, respectively [30–32]. Nevertheless, a most recent study in 19.5-month-old mice compared to juvenile mice also reported reduced osteoclastogenesis and tooth movement [33]. Overall, the age-related changes in bone remodeling during orthodontic therapy appear to contradict the general enhanced bone degradation observed in aging individuals [25, 26]. Therefore, further studies are necessary to better understand the age and aging-associated changes in orthodontic bone remodeling. In this regard, the aging-associated alterations in the mechanoresponse of PdL cells, as a relevant modulator of tooth movement, have not been sufficiently investigated.

A key challenge in comparative studies with adolescents arises from significant differences in hormone levels compared to adults and the elderly [34]. Bone remodeling processes are strongly influenced by hormones, particularly sex hormones such as estradiol and testosterone, whose levels increase during puberty and decline with age [35]. The aim of this in vitro study was to identify aging-associated changes in the mechanoresponse of PdL fibroblasts that are primarily associated with increased cellular senescence rather than hormonal changes. To this end, we focused on the comparison between adult and aged PdL fibroblasts derived from a pool of adult donors of both genders.

## Methods

### Cell culture

**Fig. 1:**
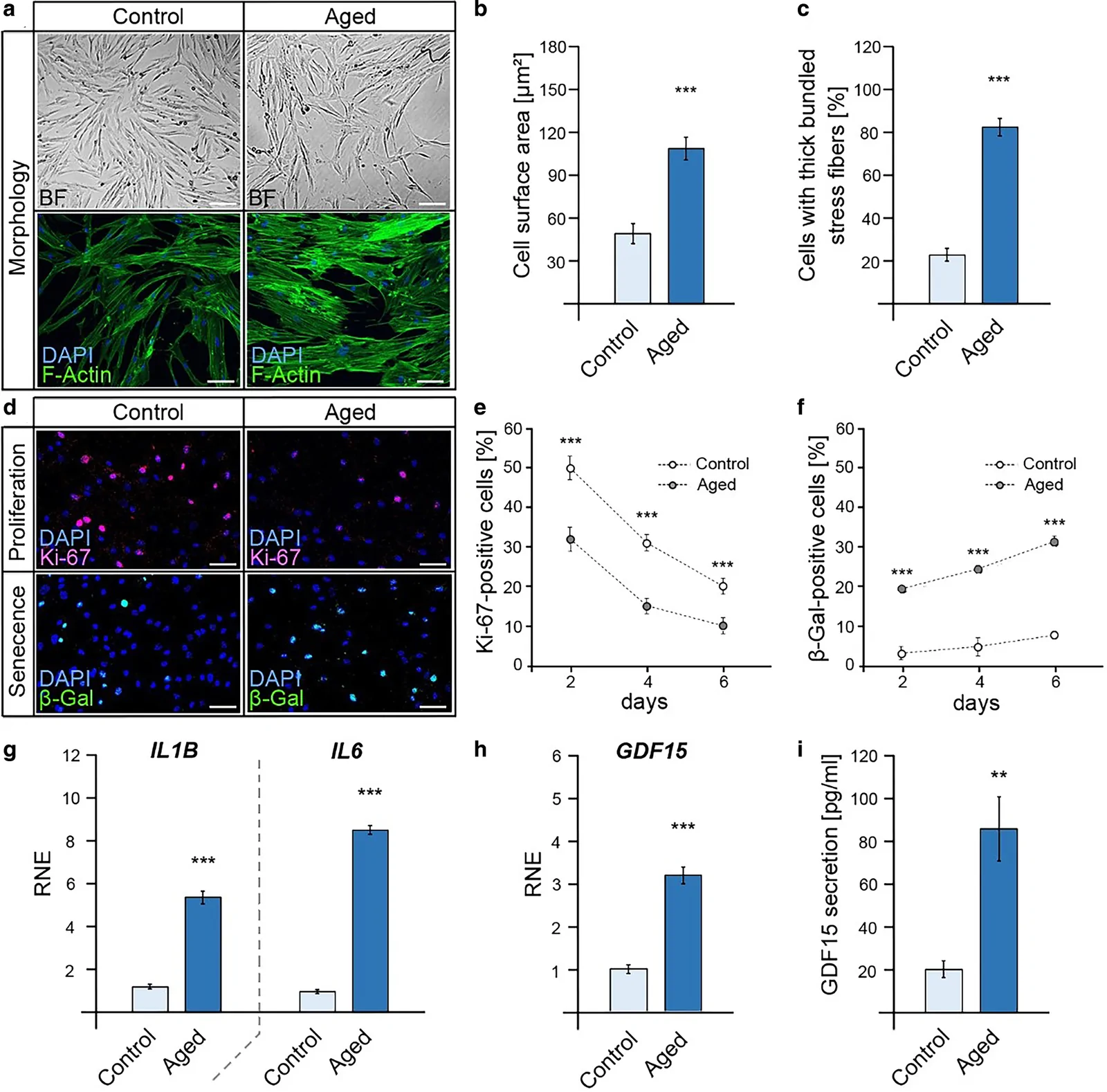
Upon frequent subcultivation, human periodontal ligament fibroblasts (hPdLFs) show a senescent phenotype with increased GDF15 levels. **a**–**c** Morphological properties of control and *in vitro* aged hPdLFs with brightfield (upper panel in **a**) and phalloidin-labeled F‑actin (green, lower panel in **a**) showing increased cell surface areas (**b**) and cells with thick bundled stress fibers (**c**). Cell nuclei are stained with 4’,6-diamidino-2-phenylindole (DAPI, blue). **d**–**f** Analysis of proliferative, Ki-67-positive cells (magenta, upper panel in **d** and **e**) and senescent, β‑galactosidase (β-Gal)-positive hPdLFs (green, lower panel in **d** and **f**). **g** Quantitative expression levels of genes encoding the pro-inflammatory cytokines IL-1β (*IL1B*) and IL‑6 (*IL6*) in aged hPdLFs. **h**–**i** Quantitative expression (**h**) and secretion (**i**) analysis of GDF15 in aged hPdLFs. ** *p* < 0.01; *** *p* < 0.001; student’s t test in **b**, **c**, **g**, **h**, and **i** and one-way analysis of variance (ANOVA) and post hoc test (Tukey) in **e** and **f**. Scale bars: 100 μm in **a **(upper panel), 25 µm in **a** (lower panel) and **d**. RNE relative normalized expression Nach häufiger Subkultivierung zeigen humane parodontale Ligamentfibroblasten (hPdLFs) einen seneszenten Phänotyp mit erhöhten GDF15 Level. **a–c **Morphologische Eigenschaften von Kontroll- und *in vitro* gealterten hPdLFs mit Hellfeld (oberes Feld in **a**) und Phalloidin-markiertem F‑Aktin (F-Actin, grün, unteres Feld in **a**) zeigen vergrößerte Zelloberflächen (**b**) und Zellen mit dichten, gebündelten Stressfasern (**c**). Die Zellkerne sind mit 4’,6-Diamidino-2-Phenylindol (DAPI, blau) gefärbt. **d**–**f** Analyse der proliferativen, Ki-67-positiven Zellen (magenta, oberes Feld in **d** und **e**) und der seneszenten, β‑Galactosidase (β-Gal)-positiven hPdLFs (grün, unteres Feld in **d** und **f**). **g** Quantitative Expressionsniveaus von Genen, die für die pro-inflammatorischen Zytokine IL-1β (*IL1B*) und IL‑6 (*IL6*) in gealterten hPdLFs kodieren. **h–i** Quantitative Expressions- (**h**) und Sekretionsanalyse (**i**) von GDF15 in gealterten hPdLFs. **p < 0,01; ***p < 0,001; Student-t-Test in **b, c, g, h** und **i **sowie einseitige Varianzanalyse (ANOVA) und Post-hoc-Test (Tukey) in **e** und **f**. Maßstab: 100 μm in **a** (oberes Feld), 25 µm in **a** (unteres Feld) und **d**. *RNE* relative normalisierte Expression

Commercially acquired human periodontal ligament fibroblasts (hPdLFs, CC-7049, Lonza, Basel, Switzerland), pooled from adults donors of both sexes, were grown in culture medium consisting of Dulbecco’s modified Eagle medium (DMEM; Thermo Fisher Scientific, Carlsbad, CA, USA) with 4.5 g/L glucose, 10% heat-inactivated fetal bovine serum (Thermo Fisher Scientific), 100 U/mL penicillin, 100 μg/mL streptomycin and 50 mg/L L‑ascorbic acid at 37 °C, under 5% CO_2_ and 95% humidity. When reaching a confluency of 75%, cells were subcultured with 0.05% Trypsin/EDTA (Thermo Fisher Scientific) and 1 × 10^6^ hPdLFs were reseeded in 20 ml culture medium in T175 culture flasks. For experiments, passages four to eight were defined as “Control” and passages 18 to 23 were defined as “Aged”. For the analysis of cellular senescence characteristics (morphology, proliferation, senescence markers), 0.5 × 10^4^ Control and Aged cells were seeded on glass cover slips in 24-well plates for six days. For analyzing RNA expression and protein secretion, 0.2 × 10^5^ Control and 2 × 10^5^ Aged hPdLFs were seeded in separate wells in a 6-well plate with either inelastic or flexible bottom and cultured to 75% confluence before further treatment. The optimal numbers of cells to be initially seeded were determined in preliminary experiments (data not shown), which was necessary due to the limited proliferation capacity of the aged hPdLFs (Fig. 1).

Nonadherent THP1 monocytic cells (DMSZ, Braunschweig, Germany) were cultured in RPMI 1640 medium (Thermo Fisher Scientific) containing 10% FBS, 100 U/ml penicillin and 100 µg/ml streptomycin at 37 °C, 5% CO_2_ and 95% humidity. Weekly passages were performed and 1.0 × 10^6^ cells were reseeded in 20 ml medium in T175 culture flasks (Thermo Fisher Scientific).

Commercially acquired human immature osteoblast (hOB, Lonza, Basel, Switzerland) were cultured in OBM™ Osteoblast Growth and Differentiation Basal Medium (Lonza), 10% heat-inactivated fetal bovine serum (Thermo Fisher Scientific), 100 U/mL penicillin, 100 μg/mL streptomycin and 50 mg/L L‑ascorbic acid at 37 °C, under 5% CO_2_ and 95% humidity.

### RNA interference by siRNA transfection

To reduce GDF15 expression levels, RNA interference was used as previously described [12, 14, 15]. To this end, cultured hPdLFs were tranfected with 50 nmol small interfering RNA (siRNA) targeting human *GDF15* mRNA (Santa Cruz Biotechnology, Dallas, TX, USA). Lipofectamine^TM^ 2000 (Thermo Fisher Scientific) was used as transfection reagent in OptiMem I‑reduced serum medium (Thermo Fisher Scientific) containing 1% penicillin/streptomycin, which was replaced by fibroblast culture medium after 5 h.

### Mechanical loading of hPdLFs

As previously reported [13, 15], a compressive force of 2 g/cm^2^ was applied for 24 h in 6‑well plates by sterile glass plates at 37 °C, under 5% CO_2_ and 95% humidity. A biaxial tensile force of 15.9% was applied as described in Steinmetz et al. [14]. To this end, spherical cap silicone stamps were placed into the bottom of flexible membrane 6‑well plates (BioFlex®, FLEXCELL®, Dunn Labortechnik, Asbach, Germany) for 24 h. Subsequently, culture supernatants of stimulated cells were isolated and cells were directly processed for RNA analysis.

### Immunofluorescent staining

To visualize cellular morphology and to analyze proliferation capacity as well as cellular senescence, immunofluorescent staining was utilized as previously described [14, 15, 36]. Briefly, cells cultured on glass cover slips were fixed for 10 min with 4% paraformaldehyde (PFA) in phosphate buffered saline (PBS). Bovine serum albumin (BSA, SEQENS, H2B-ESTER Technopole, Cedex, France) in PBS/0.1% Triton X (Sigma Aldrich, St. Louis, MO, USA) was used to block unspecific antibody binding sides. The primary antibody rabbit-anti-Ki67 (1:250, Abcam, Cambridge, UK) was used to detect the proliferation marker Ki-67. Goat anti-rabbit Alexa Fluor 488 (1:1000; Jackson ImmunoResearch, West Grove, PA, USA) was used as secondary antibody. Cell morphology was visualized by staining of F‑actin using Alexa Fluor 594 Phalloidin (Thermo Fisher Scientific). Cell nuclei were stained with 4’,6-diamidino-2-phenylindole (DAPI; 1:10,000 in PBS) and coverslips were embedded with Mowiol®4-88 (Merck Millipore, Burlington, MA, USA) for imaging.

### β-Galactosidase staining

CellEvent™ Senescence Green Detection Kit (Thermo Fisher Scientific) was used to detect cellular senescence in hPdLFs fixed with 4% PFA in PBS.

### Quantitative polymerase chain reaction (qPCR)

To analyze gene expression, RNA isolation, cDNA synthesis and quantitative PCR were performed as previously described [12]. Briefly, cells were processed with TRIzol™ Reagent (Thermo Fisher Scientific) and RNA Clean & Concentrator 5 (Zymo Research, Freiburg im Breigau, Germany) was used to isolate RNA, which was transcribed into complementary DNA (cDNA) with SuperScript IV Reverse Transcriptase (Thermo Fisher Scientific). Subsequently, quantitative expression analysis was performed using Luminaris HiGreen qPCR Master Mix (Thermo Fisher Scientific) and the qTower^3^ (Analytik Jena, Jena, Germany). Design and the analysis of primer quality, specificity and efficiency were performed as previously described [12]. Primer sequences are shown in Table 1. *RPL22* and *TBP* were used as reference genes. Internal analysis with NormFinder software (https://moma.dk/software/normfinder; version 21, accessed 10 February 2024) verified transcriptional stability of *RPL22* and *TBP* in senescent hPdLFs (data not shown). Relative normalized expression levels were calculated with the efficiency-corrected ∆∆CT method.

**Table 1:** Forward (fw) and reverse (rev) primer for quantitative expression analysis. Gene-specific exon-spanning primers are indicated in 5’–3’ direction Vorwärts- (fw) und Revers(rev)-Primer für die quantitative Expressionsanalyse. Genspezifische, exonübergreifende Primer sind in 5’-3’-Richtung angegeben

Gene name	Gene symbol	NCBI Gene ID^a^	Primer sequences
Growth differentiation factor 15	*GDF15*	9518	fw CCGAAGACTCCAGATTCCGA
			rew CCCGAGAGATACGCAGGTG
Interleukin 1β	*IL1B*	3553	fw CGAATCTCCGACCACCACTA
			rev AGCCTCGTTATCCCATGTGT
Interleukin 6	*IL6*	3569	fw CATCCTCGACGGCATCTCAG
			rev TCACCAGGCAAGTCTCCTCA
Ribosomal protein L22	*RPL22*	6146	fw TGATTGCACCCACCCTGTAG
			rev GGTTCCCAGCTTTTCCGT TC
TATA-box binding protein	*TBP*	6908	fw CGGCTGTTTAACTTCGCTTCC
			rev TGGGTTATCTTCACACGCCAAG
TNF receptor superfamily member 11b	*TNFRSF11B (*alias *OPG*)	4982	fw GAAGGGCGCTACCTTGA
			rev GCAAACTGTATTTCGCTC
TNF superfamily member 11	*TNFSF11 *(alias *RANKL*)	8600	fw ATCACAGCACATCAGAGCAGA
			rev TCACTTTATGGGAACCAGATGGG

^a^National Center for Biotechnology Information, Gene identification number

### Enzyme-linked immunosorbent assay (ELISA)

The culture supernatants isolated after final treatments of hPdLFs were examined in duplicates for protein secretion using a human GDF15 ELISA (Thermo Fisher Scientific) according to the manufacturer’s guidelines and measured with the plate reader Infinite ® M Nano (TECAN, Männedorf, Switzerland).

### Immune cell activation assay

For analyzing immune cell activation as previously described [12, 14, 15], nonadherent THP1 cells were stained with Celltracker CMFDA (15 µM, Thermo Fisher Scientific) for visualization. Aliquots of 2.5 × 10^4^ CMFDA-positive cells were stimulated with the culture supernatant of hPdLFs for 30 min in each well of a 96-well plate. After removing nonadherent cells, remaining adherent THP1 cells were fixed for 10 min with 4% PFA for imaging and cell counting.

### Osteoblast differentiation and NBT/BCIP staining

For analyzing osteoblast differentiation, 2.5 × 10^4^ immature hOBs were seeded into each well of a 96-well plate and stimulated with the culture supernatant of hPdLFs for six days with daily medium changes. After fixation with 4% PFA, cells were stained with 1‑Step™ NBT/BCIP substrate solution for 60 min and subsequently imaged. As positive control (data not shown), stimulation was performed with osteoblast growth medium additionally containing 100 nM dexamethasone and 10 mM β-glycerol phosphate.

### Osteoclast differentiation and TRAP staining

Osteoclast differentiation of THP1 cells was performed as previously described [13–15]. Briefly, 2.5 × 10^4^ cells were seeded into each well of a 96-well plate and macrophage stimulation was directly induced by a 2-day treatment with 100 ng/ml phorbol-12 myristate-13 acetate (PMA, Merck Millipore). Next, macrophage-like adherent THP1 cells were treated for six days with the culture supernatant of stressed hPdLFs with daily medium changes. As control (data not shown), macrophage-like THP1 cells were stimulated with 50 ng/ml human recombinant RANKL and 50 ng/ml human recombinant MCSF (both Thermo Fisher Scientific) in THP1 culture medium to induce robust osteoclast differentiation as previously shown [37]. TRAP staining was performed as previously described [13]. SYTO nucleic acid staining (Thermo Fisher Scientific) was performed to identify multinucleated osteoclasts.

### Microscopy, data analysis, and statistics

The inverted confocal laser scanning microscope TCS SP5 (Leica, Wetzlar, Germany) was used for imaging immunofluorescent staining. The Primovert microscope (Carl Zeiss Company, Oberkochen, Germany) was used to image THP1 cells as well as NBT/BCIP and TRAP stainings. Fiji software (https://imagej.net/Fiji; version number 1.52 p, accessed 10 January 2021) was used to analyze the number of positively stained cells. For analyzing activated osteoclast, multinucleated TRAP-positive cells were counted by overlaying TRAP and SYTO nucleic acid stainings. Graph Pad Prism 9 (https://www.graphpad.com; version 10.1.2, accessed 26 November 2021) was used for statistical analysis. All experiments were independently repeated at least three times with technical duplicates. Student’s t test and one-way analysis of variance (ANOVA) with post hoc test (Tukey) were used as statistical tests. Significance levels: */#/§ *p*-value < 0.05, **/##/§§ *p*-value < 0.01, ***/##﻿#/§§﻿§ *p*-value < 0.001.

## Results

### GDF15 is increasingly expressed and secreted by senescent PdL fibroblasts

Cellular senescence is a critical hallmark of aging and can be induced in cell culture through frequent subculturing of adherent cells by enzymatic passaging, which is therefore widely used to study aging *in vitro* [38, 39]. In the following (Fig. 1), specific cellular characteristics were analyzed to confirm cellular senescence in in vitro cultured human periodontal ligament fibroblasts (hPdLFs) of subcultures 18–23 (Aged) compared to cells of subcultures four to eight (Control).

Morphological assessments revealed a reduced fibroblastic phenotype in aged hPdLFs characterized by a less pronounced spindle-like shape, an increased cellular area and more prominent F‑actin stress fibers (Fig. 1a–c). Immunofluorescent staining of the proliferation marker Ki-67 indicated a reduced number of proliferative cells in *in vitro* aged hPdLFs (Fig. 1d, e). Furthermore, staining of β‑galactosidase (β-Gal), a commonly used senescence marker, indicated an increased number of β‑Gal-positive hPdLFs after subculturing, confirming an aged phenotype (Fig. 1d, f).

Quantitative expression analysis revealed an up-regulated expression of *IL6* and *IL1B *(Fig. 1g), encoding proteins involved in the senescence-associated secretory phenotype (SASP) in aging [18]. Additionally, further analyses demonstrated an increased expression and secretion of GDF15 by aged hPdLFs (Fig. 1h, i). In summary, our data indicate that frequent subcultivation can trigger senescence in PdL fibroblasts accompanied by elevated GDF15 levels. For further investigations, these *in vitro* aged PdL fibroblasts were used to study the adaptations of their mechanoresponse to tensile and compressive forces.

### Elevated intracellular GDF15 levels modulate the aging-associated increased response to tensile forces

**Fig. 2:**
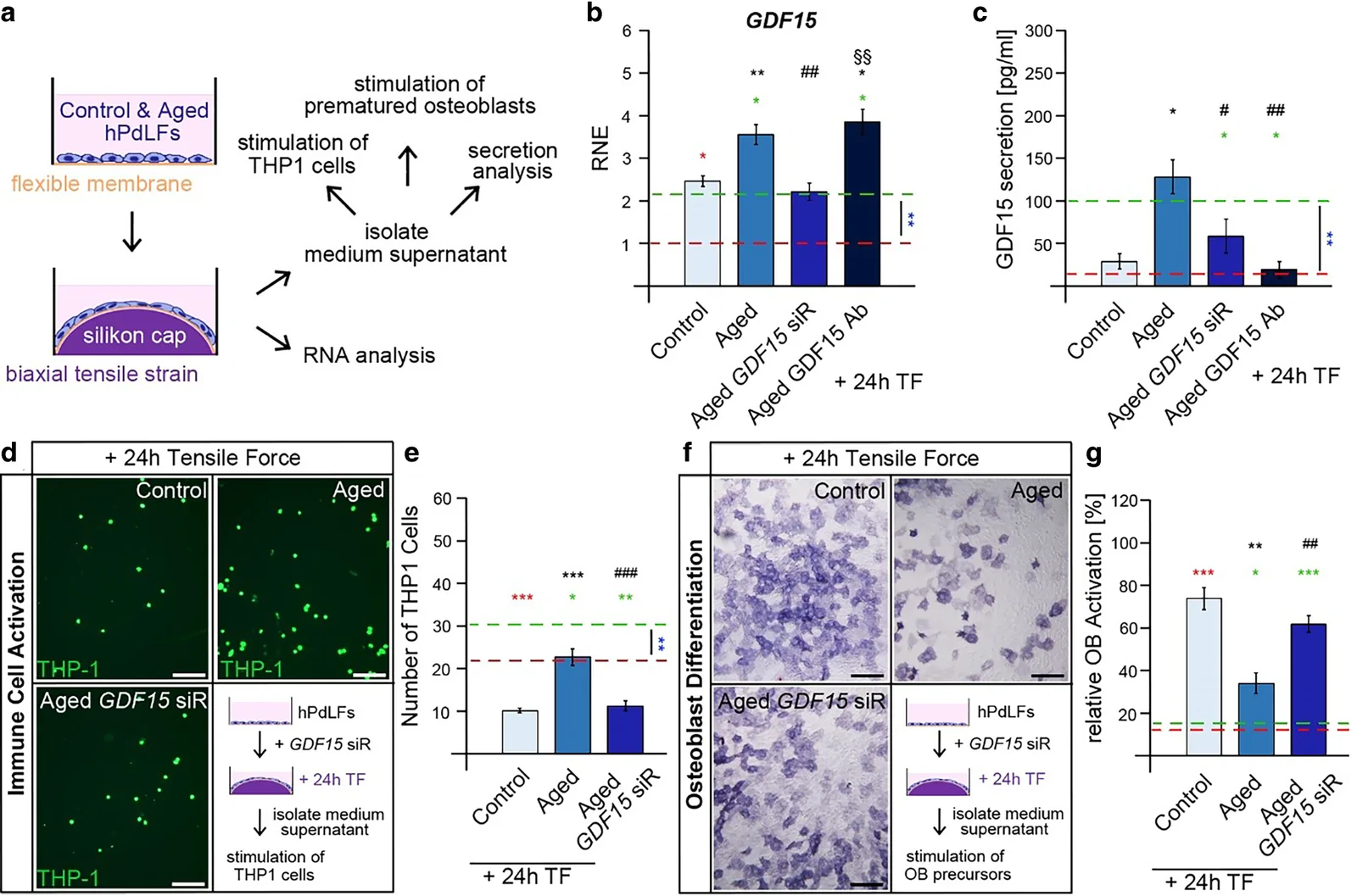
Increased intracellular GDF15 in aged hPdLFs modulates enhanced immune cell activation and decreased osteoblast differentiation after application of tensile forces. **a** Model showing the application of tensile forces (+24 h TF) to hPdLFs grown on flexible membrane plate and downstream analysis. GDF15 expression (**b**) and secretion (**c**) levels in aged hPdLFs. GDF15-targeting small interfering RNA (GDF15 siR) was used to downregulate intracellular GDF15 and the GDF15-targeting antibody (GDF15 Ab) ponsegromab was used to block extracellular GDF15. The red line indicates the unstressed control condition and red significance stars refers to this condition. The green line indicates the unstressed aged hPdLFs and green significance stars refer to this condition. Activation of monocytic THP1 cells (green in **d**) stimulated with the culture supernatant of hPdLFs analyzed as number of THP1 cells per image (**e**). Differentiation of osteoblasts (blue in **f**) stimulated with the culture supernatant of hPdLFs and analyzed as percentage of bluish area per image (**g**). * *p* < 0.5; ** *p* < 0.01; *** *p* < 0.001; One-way analysis of variance (ANOVA) and post hoc test (Tukey). Scale bars: 25 µm. OB osteoblast, RNE relative normalized expression Erhöhtes intrazelluläres GDF15 in gealterten hPdLFs moduliert eine verstärkte Immunzellaktivierung und eine verringerte Osteoblastendifferenzierung nach Anwendung von Zugkräften. **a **Modell, das die Anwendung von Zugkräften (+24 TF) auf hPdLFs, die auf einer flexiblen Membranplatte gewachsen sind, zeigt und die anschließende Analyse. GDF15-Expression (**b**) und -Sekretion (**c**) in gealterten hPdLFs. GDF15-spezifische „small interfering“ RNA (GDF15 siR) wurde verwendet, um intrazelluläres GDF15 herunterzuregulieren, und der GDF15-spezifische Antikörper (GDF15 Ab) Ponsegromab wurde verwendet, um extrazelluläres GDF15 zu blockieren. Die rote Linie zeigt die unbelastete Kontrollbedingung, rote Signifikanzsterne beziehen sich auf diese Bedingung. Die grüne Linie zeigt die unbelasteten gealterten hPdLFs an, grüne Signifikanzsterne beziehen sich auf diese Bedingung. Die Aktivierung von monozytären THP1-Zellen (grün in **d**), die mit dem Kulturüberstand von hPdLFs stimuliert wurden, wird als Anzahl der THP1-Zellen pro Bild analysiert (**e**). Differenzierung von Osteoblasten (blau in **f**), die mit dem Kulturüberstand von hPdLFs stimuliert und als Prozentsatz der bläulichen Fläche pro Bild analysiert wurden (**g**). *p < 0,5; **p < 0,01; ***p < 0,001; Einweg-Varianzanalyse (ANOVA) und Post-hoc-Test (Tukey). Maßstabsbalken: 25 µm. OB Osteoblast, RNE relative normalisierte Expression

As an important regulator of force-induced tissue and bone remodeling [12, 14], elevated levels of GDF15 in aged PdL fibroblasts may influence their mechanoresponse. To investigate their response to biaxial static strain, cultivation of hPdLFs on flexible membrane plates were performed (Fig. 2a). Elongation led to an increased expression of *GDF15* in both control and aged hPdLFs compared to the nonstressed control groups (Fig. 2b). However, *GDF15* was significantly increased in elongated aged hPdLFs compared to the stressed control (Fig. 2b). RNA silencing with a *GDF15*-specific siRNA (*GDF15 siR*) reduced this increase in *GDF15* expression to the level observed in stretched controls. In contrast, treatment with the GDF15-neutralizing antibody ponsegromab (GDF15 Ab) had no effect on *GDF15* expression levels of stressed aged hPdLFs. Further analysis of GDF15 secretion in the culture supernatant of elongated hPdLFs revealed no force-induced increase in either control or aged hPdLFs (Fig. 2c). However, GDF15 secretion was already elevated in unstressed aged hPdLFs, leading to increased levels in aged hPdLFs exposed to tensile forces, which may potentially have taken place strain-independently (Fig. 2c). Treatment with both *GDF15* siR and the GDF15 Ab resulted in decreased GDF15 secretion in stretched aged hPdLFs, restoring the levels to those observed in both stretched and unstressed controls.

As only the expression, but not the secretion of GDF15 appeared to be dependent on tensile force, we subsequently focused on the modulation of the intracellular GDF15 levels. First, we analyzed immune cell activation by examining the differentiation of nonadherent monocytic THP1 cells into adherent macrophages upon stimulation with the culture supernatant of stressed hPdLFs (Fig. 2d, e). Tensile forces led to a reduced activation of THP1 differentiation in both control and aged hPdLFs. However, even unstretched aged hPdLFs triggered significantly stronger immune cell activation than the controls, which was still detectable after application of tensile forces. The decrease in intracellular GDF15 expression by siRNA-mediated silencing compensated the enhanced number of activated immune cells in stretched aged hPdLFs.

Next, we investigated the activation of osteoblast differentiation as an important process on the tensile side during tooth movement [40]. Immature human osteoblasts were stimulated for six days with the culture supernatant of cultured hPdLFs and differentiation was assessed via alkaline phosphatase activity staining (Fig. 2f, g). While tensile forces led to increased osteoblast differentiation in both control and aged hPdLFs, the effect was less prominent in senescent fibroblasts. Interestingly, reducing intracellular *GDF15* levels by RNA interference in stretched, aged hPdLFs increased the number of osteoblast differentiation by these cells.

Together, our data suggest that elevated intracellular GDF15 levels in aged hPdLFs contribute to enhanced immune cells activation and impaired osteoblasts differentiation following tensile force application.

### Increased aging-associated responses to compressive forces are driven by extracellular GDF15 levels

**Fig. 3:**
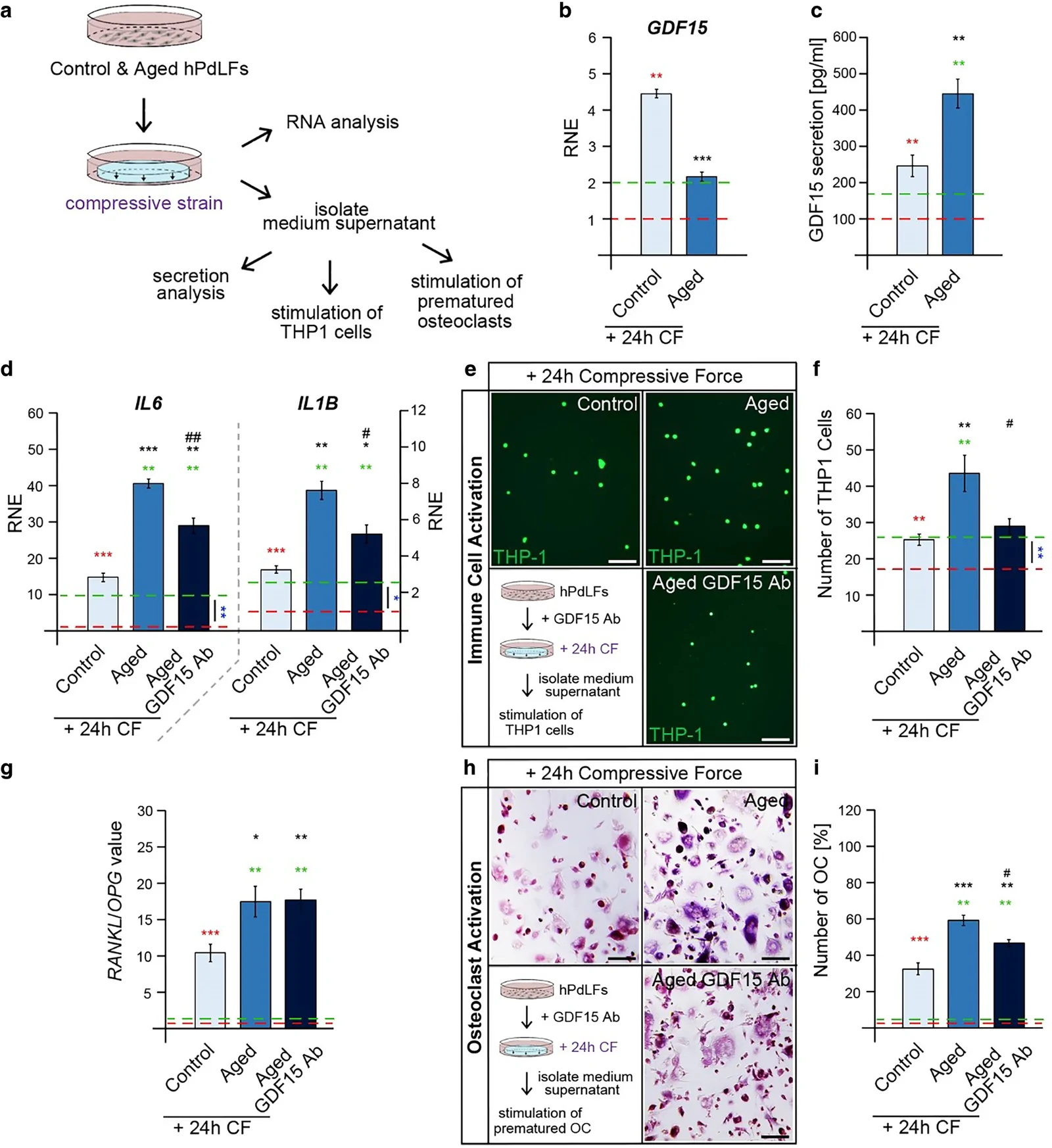
Increased extracellular GDF15 in aged hPdLFs promotes a more intense pro-inflammatory response to compressive forces with increased osteoclast activation. **a** Model showing the application of compressive forces (+24 h CF) to hPdLFs and downstream analysis. GDF15 expression (**b**) and secretion (**c**) levels in aged hPdLFs. The red line indicates the unstressed control condition and red significance stars refers to this condition. The green line indicates the unstressed aged hPdLFs and green significance stars refer to this condition. **d** Quantitative expression levels of genes encoding the pro-inflammatory cytokines IL-1β (*IL1B*) and IL‑6 (*IL6*) in compressed, aged hPdLFs. The GDF15-targeting antibody (GDF15 Ab) ponsegromab was used to block extracellular GDF15. Activation of monocytic THP1 cells (green in **e**) stimulated with the culture supernatant of hPdLFs analyzed as number of THP1 cells per image (**f**). **g** Value of *RANKL* and *OPG* expression levels in compressed, aged hPdLFs. Differentiation of osteoclasts (magenta in **h**) stimulated with the culture supernatant of hPdLFs and number of multinucleated TRAP-positive osteoclasts per image (**i**). * *p* < 0.5, ** *p* < 0.01; *** *p* < 0.001; student’s t test in **b** and **c** and one-way analysis of variance (ANOVA) and post hoc test (Tukey) in **d**, **f**, **g**, and **i**. Scale bars: 25 μm. RNE relative normalized expression Erhöhtes extrazelluläres GDF15 in gealterten hPdLFs fördert eine verstärkte pro-inflammatorische Reaktion auf Druckkräfte mit erhöhter Osteoklastenaktivierung. **a **Modell, das die Anwendung von Druckkräften (+24 h CF) auf hPdLFs zeigt und die anschließende Analyse. GDF15-Expression (**b**) und -Sekretion (**c**) in gealterten hPdLFs. Die rote Linie zeigt die unbelastete Kontrollbedingung an, rote Signifikanzsterne beziehen sich auf diese Bedingung. Die grüne Linie zeigt die unbelasteten gealterten hPdLFs an, grüne Signifikanzsterne beziehen sich auf diese Bedingung. **d **Quantitative Expressionsniveaus von Genen, die für die pro-inflammatorischen Zytokine IL-1β (*IL1B*) und IL‑6 (*IL6*) kodieren, in komprimierten gealterten hPdLFs. Der GDF15-Antikörper (GDF15 Ab) Ponsegromab wurde verwendet, um extrazelluläres GDF15 zu blockieren. Aktivierung von monozytären THP1-Zellen (grün in **e**), die mit dem Kulturüberstand von hPdLFs stimuliert wurden, analysiert als Anzahl der THP1-Zellen pro Bild (**f**). **g** Wert der *RANKL*- und *OPG*-Expressionsniveaus in komprimierten, gealterten hPdLFs. Differenzierung von Osteoklasten (magenta in **h**), die mit dem Kulturüberstand von hPdLFs stimuliert wurden, und Anzahl der mehrkernigen, TRAP-positiven Osteoklasten pro Bild (**i**). *p < 0,5, **p < 0,01; ***p < 0,001; Student-t-Test in **b** und **c**, einseitige Varianzanalyse (ANOVA) und Post-hoc-Test (Tukey) in **d, f, g **und** i**. Scale bars: 25 μm. RNE relative normalized expression Maßstab: 250 μm. RNE relative normalisierte Expression

GDF15 is a relevant mediator of compression-induced inflammation and osteoclast activation by PdL fibroblasts [12, 15, 16]. Therefore, we next investigated its role in the response of senescent hPdLFs to compressive forces (Fig. 3a). While intracellular GDF15 expression in control cells was enhanced by compression, aged hPdLFs did not exhibit such an increase (Fig. 3b). However, GDF15 secretion was elevated in response to compressive forces in both control and aged hPdLFs, with significantly higher extracellular levels in the senescent cells (Fig. 3c). We therefore utilized the GDF15-specific antibody ponsegromab (GDF15 Ab) to functionally reduce these elevated extracellular GDF15 levels.

To assess inflammatory responses, we examined the expression of genes encoding the pro-inflammatory cytokines IL‑6 (gene: *IL6*) and IL-1β (gene: *IL1B*) (Fig. 3d). We detected a force-related enhanced gene activity for *IL6 *and *IL1B* in both the control and the aged hPdLFs. However, the compressed senescent cells showed significantly higher expression levels of *IL6* and *IL1B* compared to the stressed control, a difference that was also evident under unstressed conditions. Treatment with the GDF15 Ab attenuated this elevated expression of *IL6* and *IL1B* in compressed aged hPdLFs. Functionally, analysis of immune cell activation revealed an aging-associated increase in THP1 differentiation after application of compressive forces compared to the stressed control (Fig. 3e, f). Reducing extracellular GDF15 in stressed aged hPdLFs via the GDF15 Ab mitigated this up-regulated immune cell activation.

Next, we assessed the *RANKL*/*OPG *ratio as a relevant predictor of osteoclast activation (Fig. 3g). Compressive force elicited a significant increase in the *RANKL*/*OPG *ratios in both the control and senescent hPdLFs, with a more pronounced elevation in aged cells. Blocking extracellular GDF15 with ponsegromab had no significant effect on this ratio. Subsequently, we investigated the impact of aging on the force-induced osteoclast differentiation by hPdLFs (Fig. 3h, i). Preactivated THP1 macrophages were stimulated with the culture supernatant of hPdLFs and the resulting number of multinucleated TRAP-positive osteoclasts was analyzed. Compressive forces enhanced osteoclastogenesis, which was significantly up-regulated by aged hPdLFs. Blocking elevated extracellular GDF15 levels in compressed aged hPdLFs reduced the number of activated osteoclasts. However, activation remained higher compared to the stressed control.

Overall, increased extracellular GDF15 levels appear to drive the aging-associated increase in immune cell and osteoclast activation by compressed PdL fibroblasts.

## Discussion

In this *in vitro* study, aging-associated adaptations in the mechanoresponse of periodontal ligament fibroblasts (PdLFs) were investigated, exploring a potential regulatory role of GDF15 in these senescence-related changes. Along with a reduced proliferative capacity, an increased expression of senescence-associated markers and an altered morphology, aged PdL fibroblasts exhibited major adaptations in their response to applied mechanical forces. In addition to an increased pro-inflammatory response of aged cells, their force-dependent activation of osteoblasts and osteoclasts was reduced. Modulating elevated intra- and extracellular GDF15 levels in senescent PdL fibroblasts partially balanced their hyperinflammatory response as well as aging-associated changes in the activation of bone-remodeling cells. Given that efficient bone remodeling is essential for controlled orthodontic tooth movement, these findings suggest that aging may compromise the orthodontic treatment, particularly in terms of periodontal stability and bone remodeling. In summary, our *in vitro *data indicate significant aging-induced alterations in the mechanoresponse of PdL fibroblasts, partially modulated by GDF15.

Similar to other studies [38, 39], we induced replicative cellular senescence in hPdLFs by repeated enzymatic subculturing, resulting in senescent hPdLFs. Their phenotype was characterized by a decreased proliferative capacity, increased expression of the senescence-associated β‑galactosidase and morphological changes from a spindle-shaped to a more enlarged and flattened appearance [38, 39, 41]. In addition, aged hPdLFs showed increased thickness of stress fibers, an important component of the cytoskeleton, likely resulting from senescence-induced reorganization of actin–myosin structures and altered levels of associated proteins, as previously shown for skin fibroblasts [42]. These highly dynamic actin fibers are connected to the extracellular matrix via various actin-associated cytoskeletal proteins, signaling molecules, and integrins. This enables the detection of extracellular mechanical cues leading to the adaptation of cellular morphology and gene expression through their linkage to nuclear lamins via the LINC complex [43–45]. This is of particular relevance as significant changes in the transcriptome of aged PdL fibroblasts have been reported [45].

We detected increased expression levels of genes encoding the SASP-associated cytokines IL‑6 and IL-1β, as well as GDF15. The distant member of the bone morphogenic protein (BMP) family and the transforming growth factor-beta (TGF-β) superfamily were shown to be significantly increased with age and has been linked to a variety of age-related diseases [46, 47]. Therefore, GDF15 was recently proposed as SASP and biomarker for aging [17]. Interestingly, *in vitro* stimulation of hPdLFs with recombinant human GDF15 (rhGDF15) directly promoted cellular senescence, as indicated by increased expression of β‑galactosidase and p21^Waf1/Cip1^ [13]. This suggests a complex process in which GDF15 may play an important role as an aging-promoting factor and an effector protein.

The pleiotropic modulator GDF15 elicits both anti- and pro-inflammatory functions highly depending on the cellular context as well as on the specific stress signals [48]. In the mechanoresponse of PdL fibroblasts, GDF15 was reported repeatedly to promote cytokine secretion and immune cell activation [12–15]. In line with this, we detected increased GDF15 expression and secretion correlating with enhanced pro-inflammatory cytokine levels and up-regulated differentiation of monocytic THP1 cells due to mechanical stimulation. However, aged PdLFs showed significantly higher GDF15 levels and a more pronounced pro-inflammatory response, although this was to some extent dependent on the force type. This seems to be consistent with the reported pro-inflammatory state of aging cells in general [19] and PdL cells in particular [49, 50]. Given that chronic inflammation in the periodontal ligament is associated with an increased risk of root resorption and attachment loss, the exaggerated inflammatory response in aged PdLFs could contribute to periodontal breakdown during orthodontic treatment. Nevertheless, there seems to be a force-dependent adaptation in the mode of action. While in aged PdL fibroblasts both GDF15 expression and secretion were increased after application of tensile forces, we observed only a higher secretion of GDF15 in compressed cells. Aging might affect force-type-specific gene expression profiles and protein release, potentially due to cytoskeletal changes altering cellular mechanosensitivity [45].

GDF15 is synthesized as a precursor protein and only secreted after disulfide-linked dimerization [51]. We recently reported that in elongated PdL fibroblasts, GDF15 may act intracellularly via IL-37 modulating their osteoblastic phenotype and subsequent osteoclast activation [14]. However, secreted but inactive GDF15 can also be bound to extracellular matrix (ECM) components for storage, and stress-dependent activation of specific matrix metalloproteinases (MMPs) can lead to GDF15 release from the ECM [52, 53]. The increased expression and activation of ECM-degrading MMPs observed with aging [54] may be further enhanced by compressive forces, which generally also stimulate a pronounced ECM activation [55]. This could lead to an increased release of GDF15 in compressed, aged hPdLFs without up-regulating its intracellular expression.

While bone density increases from adolescent to adulthood, further aging impacts alveolar bone remodeling by decreasing turnover rates, thus, compromising structural integrity and increasing the risk of bone loss [25, 26]. The underlying mechanisms are complex and potentially rely on changes in the differentiation and activity of bone-remodeling cells, including but not limited to osteoblasts and osteoclasts [56]. In comparison between juvenile and adult stages, reduced osteoblastogenesis has been frequently reported in orthodontic treatment of patients and in experimental OTM animal studies [28, 29, 33]. In line with this, we detected a reduced activation of osteoblast differentiation initiated by elongated, senescent PdL fibroblasts, emphasizing that aging limits bone assembly. This also appears to be in accordance with the age- and aging-associated impairments of osteoblastogenesis, which has been similarly reported [57]. The formation of new alveolar bone material, primarily promoted at the tensile side, is critical for tooth anchorage during orthodontic therapy [40]. Given the age and aging-associated decline [57], one might speculate that this may contribute to the increased risk of attachment loss and tooth loss in older patients. However, we demonstrated that aging-associated intracellular GDF15 signaling might contribute to the limited ability of elongated PdL fibroblasts to activate osteoblast differentiation. Besides potential direct effects of GDF15 on osteoblasts [58, 59], intracellular signaling pathways of immature GDF15 have also been described to modulate osteoblast-regulating factors in PdLFs [14].

When comparing adolescent and adult orthodontic patients as well as experimental OTM animals, several studies have reported decreased osteoclast differentiation and activity [1, 27, 28]. However, aging is generally associated with decreased bone density and increased osteoclast activity, leading to various aging-associated bone disorders, including osteopenia, osteoporosis, and osteoarthritis [60]. We detected enhanced *RANKL*/*OPG* ratios in aged PdL fibroblasts when stimulated with compressive forces. This seems to be in accordance with a variety of different studies particularly showing increased RANKL levels in several senescent cell types due to cellular stress, including cells isolated from aged mouse bone marrow [61], senescent human osteoblasts [62], and osteocytes [63]. However, the enhanced osteoclast differentiation mediated by secreted factors from compressed, aged PdL fibroblasts supports our hypothesis that aging and associated cellular changes may exacerbate compressive force-induced bone resorption. This seems plausible in view of the generally increased osteoclast activity and subsequent bone loss in the elderly [25, 26]. We further demonstrated that the increased extracellular GDF15 levels secreted by compressed, aged PdL fibroblasts partially contribute to osteoclast hyperactivation. This is consistent with studies demonstrating a direct influence of GDF15 on osteoclast differentiation [58, 64].

For several reasons, correlations with previously published studies examining age-specific changes in osteoclast activation by orthodontic forces are limited. Most importantly, these studies consistently compare adolescent and adult subjects [27, 28], but did not include aged individuals based on the general age classifications for humans and rodents [30–32]. From a clinical perspective, this distinction is crucial, as aging-associated changes in periodontal and bone remodeling mechanisms are likely to affect the predictability and efficacy of orthodontic interventions in elderly patients. For the interpretation of our findings, it is essential to distinguish between age-related and aging-associated changes. There is considerable evidence regarding the influence of sex hormones during orthodontic therapy, which impacts PdL cell properties and functionality as well as bone remodeling [65, 66]. Moreover, a correlation between hormonal changes with GDF15 levels was previously reported [67, 68]. Considering that the levels of sex hormones initially increases significantly in adolescence and then decrease with age [35], this could be a key factor contributing to the differences between adolescent, adult, and elderly patients. In this study, we investigated aging-associated differences independently of hormonal changes by comparing *in vitro* cultured and senescent PdL fibroblasts derived from a pool of adult human donors of both sexes. Thus, the detected differences in the mechanoresponses of those cells can be attributed to the aging process of the senescent PdL cells. However, this can also be seen as a limitation of the study, as this can only provide insights into a section of the overall events underlying the complex aging processes. Future *in vivo* studies should include adolescents in addition to adults and the elderly to allow the identification of age-related changes while also considering sex differences. However, such an approach was beyond the scope of our study.

## Conclusion

Aging was demonstrated to alter the mechanobiological response of periodontal ligament cells. Reduced osteoblast activation and enhanced osteoclast-induced resorption may impair alveolar bone remodeling and increase the risks of root resorption, attachment loss, and tooth instability. Aging-associated elevation in GDF15 levels exacerbate inflammatory and resorptive responses to mechanical forces and may further complicate treatment. These findings underscore the need for personalized orthodontic strategies in older patients, potentially targeting modulators such as GDF15 to optimize bone remodeling. Addressing these aging-related changes is crucial for improving orthodontic care for the elderly.

## Data Availability

The datasets of this study are available upon reasonable request from the corresponding author.
